# Genome characterization of Rift Valley fever virus isolated from cattle, goats and sheep during interepidemic periods in Kenya

**DOI:** 10.1186/s12917-024-04161-1

**Published:** 2024-08-23

**Authors:** Amos A. Onwongá, Samuel O. Oyola, John Juma, Samson Konongoi, Richard Nyamota, Reuben Mwangi, Collins Muli, Paul Dobi, Bernard B. Bett, Juliette R. Ongus

**Affiliations:** 1 Jomo Kenyatta University of Agriculture and Technology (JKUAT), Nairobi, Kenya; 2 Department of Veterinary Services, Ministry of Agriculture Livestock and Fisheries, Nairobi, Kenya; 3 International Livestock Research Institute (ILRI), Nairobi, Kenya; 4 Kenya Medical Research Institute, Nairobi, Kenya

**Keywords:** Rift Valley fever virus, Genome, Reassortment, Epidemics, Virus and Segment

## Abstract

Rift Valley fever virus (RVFV) is a mosquito-borne RNA virus of the *Phlebovirus* genus in the *phenuviridae* family. Its genome is trisegmented with small (S), medium (M) and large (L) fragments. In nature, the virus exists as a single serotype that is responsible for outbreaks of Rift Valley fever (RVF), a zoonotic disease that often occurs in Africa and the Middle East. RVFV genomes are thought to undergo both recombination and reassortment and investigations of these events is important for monitoring the emergence of virulent strains and understanding the evolutionary characteristics of this virus. The aim of this study was to characterize the genomes of RVFV isolates from cattle, sheep, and goats collected during an interepidemic period in Kenya between June 2016 and November 2021. A total of 691 serum samples from cattle (*n* = 144), goats (*n* = 185) and sheep (*n* = 362) were analysed at the Central Veterinary Laboratories. The competitive IgM-capture ELISA, was used to screen the samples; 205 samples (29.67%) tested positive for RVFV. Of the 205 positive samples, 42 (20.5%) were from cattle, 57 (27.8%) from goats, and 106 (51.7%) from sheep. All the IgM-positive samples were further analyzed by qPCR, and 24 (11.71%) tested positive with Ct values ranging from 14.788 to 38.286. Two samples, 201808HABDVS from sheep and 201810CML3DVS from cattle, had Ct values of less than 20.0 and yielded whole genome sequences with 96.8 and 96.4 coverage, respectively. There was no statistically significant evidence of recombination in any of the three segments and also phylogenetic analysis showed no evidence of reassortment in the two isolated RVFV segments when compared with other isolates of different lineages from previous outbreaks whose genomes are deposited in the GenBank. No evidence of reassortment leaves room for other factors to be the most probable contributors of change in virulence, pathogenicity and emergence of highly virulent strains of the RVFV.

**Supplementary Information:**

The online version contains supplementary material available at 10.1186/s12917-024-04161-1.

## Introduction

Rift Valley fever virus (RVFV) is a mosquito-borne RNA virus that belongs to the *Phlebovirus* genus in the *Phenuiviridae* family [1, 2]. Its genome is trisegmented with small (S), medium (M) and large (L) fragments. It is a vector borne virus which together with other environmental, vector and host factors cause outbreaks of Rift Valley fever (RVF) in humans and livestock in Africa and the Middle East. It causes abortion in susceptible pregnant animals irrespective of the gestation period and high mortality in new-borns [3]. Outbreaks do not invariably involve a single genotype of virus but can result from intensive transmission of multiple strains already circulating in endemic areas [4, 5].

RVFV transmission is mainly by the *Aedes* and *Culex* mosquito species in ruminants and camels. Inter-epidemic periods can last for 5 to 15 years or between 3 and 5 years in some endemic areas [6]. Studies in Uganda [7] have provided evidence of RVFV circulation among healthy animals during these periods. In such cases, the virus will be detected in the absence of obvious clinical manifestations like abortions.

Epidemics in humans are unpredictable and often involve several countries in the affected region at the same time [8]. In humans, transmission is through contact with infected body fluids or blood, ingestion of unpasteurized or uncooked by-products of infected animals, or through inhalation of aerosols produced during the slaughter of infected animals. It can also occur from the bites of infected mosquitoes, mainly by *Aedes* and *Culex,* but also by the *Anopheles* or *Mansonia* species. Other blood-feeding vectors, such as flies and ticks, have been identified to also spread this virus [9]. In humans, the incubation period is 4 to 6 days, followed by disease that manifests on a spectrum, from asymptomatic or mild illness with headache, fever, and muscle and joint pains to severe illness associated with haemorrhagic fever, encephalitis, or ocular disease [10]. A complete recovery may take weeks. However, immunocompromised patients may die 3 to 6 days after the onset of symptoms. Up to 10% of patients may also suffer from partial or complete vision loss, which might begin immediately after the onset of symptoms [11]. The mortality rate for humans can be as low as 2% or as high as 45% [12].

There have been a series of major outbreaks such as the Egyptian RVF outbreak in 1977 resulting in 200,000 human infections and 600 deaths, Mauritania outbreak in 1987 where 200 human deaths occurred, Madagascar RVF outbreak in 1991 and in eastern Africa RVF outbreak of 1997–1998 which led to 89,000 infections and more than 500 deaths reported in Kenya, Tanzania, Somalia [13]. The last major outbreak in Kenya happened in 2006–2007 with minor outbreaks in 2014 and 2018 [14]. Because RVF epidemics are recurrent, hundreds of thousands of livestock are killed, causing significant economic losses to rural people who are seriously affected in terms of food security and household nutrition through direct and indirect losses to livestock production [1, 15].

In Kenya, RVFV persists in the environment through vertical transmission in mosquitoes and horizontal transmission by mosquitoes among animals in which the principal hosts have not been identified [16]. During the dry season, infections are low, but the RVFV is maintained by vertical transmission within the *Aedes* mosquito population [17]. During the heavy rainfall season, infection rates shoot up when mosquito eggs hatch in floodwater. This results in the amplification of vectors that feed on viremic ruminants and humans [18].

In nature, RVFV exists as a single serotype that yields differences in virulence during outbreaks. Theoretically, the virus may increase diversity by undergoing both genetic recombination and natural reassortment from its segmented genome. The latter requires distinct strains to simultaneously co-infect the same cell and reshuffle their segments [19]. This has been demonstrated experimentally in tissue cultures and in mosquitoes that were dually infected [20]. But does it happen in nature as part of evolution and natural selection?

In Kenya there have been studies on RVFV, pathogenesis, molecular epidemiology, vectors, diagnostics, prevention and also determine the phylogenetic relationships of isolates involved in particular outbreaks [21]. However, these studies and reviews have mainly involved isolates from outbreaks before 2016 [22–26] and therefore there was need to continue similar studies on isolates from 2016 to 2021. However, to unravel the evolutionary characteristics of RVFV, monitoring molecular events through genome characterization is necessary as part of the wider molecular epidemiology of RVFV. It was therefore important to genetically analyse the samples that were collected between 2016 and 2021 to try and check whether there have been any significant changes in the three segments S, M and L of the RVFV strains currently circulating in Kenya and which have not been genomically analysed before. Importantly, there is need to continually monitor RVFV genomes to detect any genetic changes within the virus genome as part of genome surveillance.

The aim of this study was to determine RVFV infection status of cattle, sheep and goats during an interepidemic period between 2016 and 2018 and characterize the genomes of RVFV isolated.

## Materials and methods

### Study set-up

This study was conducted on RVF viruses in serum samples from livestock that were referred to Central Veterinary Laboratories (a national reference laboratory for animal diseases) in Kenya from all over Kenya during the interepidemic period, between June 2016 and November 2021. Following a Cochran sample size calculation, 691 blood samples were conveniently collected from cattle (*n* = 144), goats (*n* = 185), and sheep (*n* = 362) (Fig. S1), which met the case definition for Rift Valley fever infection with a temperature > 37.5 °C. The serum samples were stored at -80 °C at the Central Veterinary Laboratories until routine RVF diagnostic testing was done. All the work that involved the virus culture and nucleic acid handling was done at the International Livestock Research Institute (ILRI) in a BSL3 laboratory under a Class 2A biosafety cabinet. The results were tested using the Chi-square statistical method at *P *= 0.05 and 2 degrees of freedom.

### Screening of serum samples with IgM antibody capture ELISA

Serum samples from livestock with recent infections were identified using the ID Screen® Rift Valley Fever IgM Capture Kit (Innovative Diagnostics, Grabels, France) as per the manufacturer’s instructions. Briefly, samples and controls were added in duplicate to adjacent even and odd-numbered wells pre-coated with anti-bovine-ovine-caprine IgM polyclonal antibodies. Plates were washed and the RVFV nucleoprotein was added to the even-numbered columns only. The RVFV nucleoprotein is fixed to the anti-RVFV IgM antibodies present in the sample and captured on the plate. After washing off excess RVF nucleoprotein, an anti-RVFV nucleoprotein monoclonal antibody conjugated to HRP was added and fixed to the nucleoprotein previously captured on Rift Valley fever virus IgM antibodies. The excess unbound conjugate was washed off and the substrate solution (TMB) added. The resulting coloration depended on the quantity of specific antibodies present in the sample tested. The source of RVFV nucleoprotein added to the plates was a recombinant protein and was produced by being expressed by E. coli BL21 containing the RVFV-NP expression plasmid.

### Extraction of total RNA from serum samples that tested positive for RVFV IgM

Total RNA was extracted from serum using the TANBead Nucleic Acid Extraction Kit (OptiPure Viral Auto Plate/Auto Tube (665) (TANBead Technology, Taiwan) according to the manufacturer’s instructions. Briefly, an aliquot of 300µL of serum was mixed with TANBead lysis buffer in the reagent tubes held in a plate and incubated at room temperature for 10 min. The lysate was then placed into the TANBead machine, where the extraction process proceeded automatically until the purified nucleic acid was obtained.

### Detection of the RVFV L-Segment by RT-qPCR

For each sample, the one-step RT-qPCR mix was prepared as follows: Nuclease-free water (4.75 µl), oligonucleotides 0.75 µl, and Probe 7.5 µl (Table S1), 13 µl of PCR reaction mix was added to a 96-well PCR plate for each sample that was to be assayed, followed by 2 µl of the extracted RNA to give a final reaction volume of 15 µl, and the plate was covered with the plate sealer and centrifuged for 1 min in a suitable centrifuge to mix and settle the contents of each well. The plate was then placed in the QuantStudio 5™ qPCR machine from Thermo Fisher Scientific Applied Biosystems, and the cycle reactions were set as volume 15uL, cover temperature 105.0 °C. The holding stage was held in two steps as follows: step 1 at 50.0 °C for 10 min, step 2 at 95.0 °C for 2 min, while the PCR stage was also a two-step process with step one at 95.0 °C for 3 min and step 2 at 60.0 °C for 30 s for 40 cycles, and all the steps took 1.6 cycles per second. Samples with Ct values under 20.0 underwent direct sequencing, while those with Ct values above 20.0 underwent viral culture enrichment prior to whole genome sequencing.

### Sequencing of the isolated RVV genomes

Each of the three RVFV segments S, M and L underwent full amplification before sequencing. cDNA was prepared using the Lunascript RT supermix (New England Biolabs, Hitchin, United Kingdom) in a 10µL reaction according to manufacturer instructions. Then a multiplex PCR was set up in two separate reactions with pooled primers. Two Eppendorf tubes were labelled primer pool 1 and prime pool 2. An equal volume of odd-numbered primers was added to primer pool 1 to produce 38 primer pools, while even-numbered primers were added to primer pool 2 to produce 36 primer pools. The primer pools were then diluted at a ratio of 1:10 with TE buffer to a working concentration of 10 µM, which underwent the following reactions: 98 °C for initial denaturation for 30 s, 35 cycles of denaturation at 95 °C for 15 s, annealing at 65 °C for 5 min, and finally holding at 4 °C.

Library preparation of the samples was performed using the NEBNext Ultra II DNA library preparation kit (New England Biolabs, Ipswich, MA, USA). The AMPure XP purification beads (Beckman Coulter, Wycombe, UK) were used to clean the PCR products, and Qubit 2.0 (Thermo Fisher Scientific, Waltham, MA, USA) was used to measure their size. The NEBNext Ultra II DNA Library Preparation Kit (New England Biolabs, Hitchin, UK) was used for end repair and adapter ligation. AMPure XP beads were used to clean and connect the adapters. Next, the NEBNext Ultra II Q5 Master Mix, TruSeq Index Primers (i7), and Universal PCR Primers (i5) were used in a 50μL reaction for 15 cycles of PCR. Equal volumes were pooled and cleaned. Using AMPure XP beads, purification was done and quantified. Denaturation and dilution of the pooled libraries were done before loading onto an Illumina sequencing instrument according to the manufacturer’s instructions.

### Phylogenetic analysis of the RVFV genome isolates from cattle, sheep, and goats

The nucleotide sequence data was assembled into continuous contigs using BWA 7.1.1 software and protein sequences were derived from the encoding regions using bioinformatics tools. BLAST search was done to establish the relationship between the sequenced RVFV isolates and other RVFV sequences deposited in NCBI GenBank®. While ensuring all the RVFV lineages were included in the analysis, the sequences with the highest scoring homologies on BLAST for each segment were separately concatenated into a single FASTA file and then analysed by multiple sequence alignment using the MUSCLE application within the Molecular Evolutionary Genetic Analyser (MEGA) software version 11, and phylogenetic trees derived using Maximum Parsimony from 1000 bootstrap replicates. The best-fitting trees were selected.

### Identification of S, M, and L genome segment recombinants of the RVFV isolates

Recombination within the S, M, and L genome segments was detected from multiple sequence alignments fed into the RDP, GENECONV, and MAXCHI tools within the RDP software version 5 at a P-value of 0.05. A total of 100 sequences each for S, M, and L complete genome segment sequences of RVFV isolates were selected from GenBank by running a BLAST search and aligned using MUSCLE in MEGA software version 11. The RDP version 5 software was opened and settings adjusted as follows: sequence adjusted to linear, RDP, GENECONV, and MAXCHI recombination methods were selected since the sequence dataset was large (> 50 sequences), overlapping events were disentangled, and the RUN icon was pressed.

### Detection of natural reassortment events in the isolated RVFV genomes

Using phylogenetic trees generated by the maximum likelihood method for the S, M, and L segments, natural reassortment was detected by observing the clustering of the S, M, and L segments of the two RVFV isolates with other genome segments extracted from the NCBI GenBank.

## Results

### Active RVFV infections in the study population of cattle, sheep, and goats

Of the 691 samples analysed, 205 (29.67%) tested positive on IgM capture ELISA. Of the 205 IgM-positive samples, 42 (20.5%) were from cattle, 57 (27.8%) from goats, and 106 (51.7%) from sheep. Overall positivity was 29.67% with species positivity being 29.2% for cattle, 30.8% for goats and 29.3% for sheep. A statistical analysis of these results using a Chi square test showed that the pravelences varied significantly by species (*P *< 0.05). All the IgM-positive samples were further analysed by RT-qPCR to confirm the presence of an active infection at the time the samples were collected. Of the 205 IgM-positive samples, 24 (11.71%) tested positive on RT-qPCR, with 12.5% from cattle, 8.3% from goats, and 70.8% from sheep. Of the RT-qPCR positive samples, 18 (75%) were cases from the interepidemic period and the remaining 6 were from a minor outbreak that occurred between June 2018 and September 2018 (Fig. S2) that fell smirk in the middle of the study window. The RT-qPCR positive samples gave Ct values ranging from 14.788 to 38.286. A statistical analysis of these results based on the Chi square test also showed that the infection levels were significantly different between species (*P *< 0.05).

### Sequencing of the isolated RVFV genomes

Only two samples (201808HABDVS from sheep and 201810CML3DVS from cattle) with a Ct value of ≤ 20.0 produced complete genome sequences 96.8 and 96.4 coverage, respectively following sequencing. We did not obtain 100% coverage because of possible low virus particles in the RNA template for the samples as indicated by Qubit (ThermoFisher Scientific. One hundred sixty-eight third avenue Waltham, MA USA) concentration readings. In addition, low coverage regions were the ends of the genome which are not amplified perfectly by the amplicon multiplex primers.

The complete genome sequences of these two RVFV strains were deposited in GenBank and assigned accession numbers (Table S2). And even though the other 22 samples with Ct values > 20 underwent virus enrichment by virus culture and showed distinct cytopathic effects (CPE), those with both suspect and clear CPEs were retested by RT qPCR after virus enrichment but there was no significant difference in Ct values between the raw serum samples and when the serum was enriched as none of the samples had a Ct Value of < 20 and hence they did not yield whole genome sequences for the S, M, and L segments on sequencing.

### Phylogenetic analysis of the sequenced S, M, and L segments of the RVFV isolates

The S segment encodes the nonstructural protein (NSs), which acts as a major determinant of virulence by antagonizing interferon beta gene expression. This protein was found to be the conserved region for the S segment. Both the 201808HABDVS from sheep and the 201810CML3DVS from cattle had a conserved region that was 710 amino acids long. For the 201808HABDVS strain, it started at amino acid number 25 and ended at 735, and for the 201810CML3DVS, it started at amino acid position 47 to 757. From the analysis of alignment data for other sequences obtained from NCBI BLAST, the S segment for 201808HABDVS had a 99.76% identity with the first five S segment sequences of RVFV isolates 2,001,707,023, 20,190,275, 201,902,750, 201,900,879, and 201,900,880, all from Uganda, while the lowest percentage identity was 97.85 for the last 23 sequences. The 201810CML3DVS from cattle had a highest percentage identity of 97.90% with the RVFV isolate 201,902,734 from Uganda and a lowest percentage identity of 97.79 with the RVFV isolate SPU/09 from the Republic of South Africa.

Phylogenetic tree for S Segment Fig. S3 constructed from a total of 33 sequences of different lineages from NCBI Genbank showed both isolates OP158212 and OP158209 both highlighted in yellow belong to lineage C and clustered with other isolates from previous outbreaks determined to be in lineage C.

The M segment of RVFV encodes four conserved gene products: two viral envelop glycoproteins G1 and G2, a glycosylated 78-kDa protein, and a non-glycosylated 14-kDa protein. The G1 glycoprotein for both isolates, 201808HABDVS from sheep and 201810CML3DVS from cattle, was 1601 amino acids long, starting at amino acid position 462 to 2063. The G2 C-terminal glycoprotein was 512 amino acids long, starting from the amino acid position 3075 to 3587. The G2 glycoprotein was 968 amino acids long, starting at amino acid position 2067 to 3035, and the Ns-M (nonstructural M protein) region was 449 amino acids long, from amino acid position 3 to 452.

From the analysis of alignment data for other sequences obtained from NCBI BLAST, the M segment for 201808HABDVS from sheep had a 99.85% identity with the first three sequences of RVFV isolates 2,001,707,023, 201,902,753, and 201,902,750 all from Uganda, while the lowest percentage identity was 97.50 with the last RVFV strain Kenya83(24,445) M complete sequences from Kenya. The 201810CML3DVS from cattle had a highest percentage identity of 99.97% with the first three RVFV isolates, 201,707,023, 20,190,275, and 201,902,750 M segment complete sequences, all from Uganda, and a lowest percentage identity of 97.58% with the RVFV isolate Kenya83(21,445) M segment complete sequence from Kenya.

Phylogenetic tree for M Segment Fig. S4 constructed from a total of 30 sequences of different lineages from NCBI Genbank showed both isolates OP158210 and OP158213 both highlighted in yellow belong to lineage C and clustered with other isolates from previous outbreaks determined to be in lineage C.

The L segment of both 201808HABDVS from sheep and 201810CML3DVS from cattle had a region that was 2087 amino acids long and encoded RNA-dependent polymerase. For 201808HABDVS from sheep, it started at amino acid position 1801 and terminated at 3888, while for 201810CML3DVS from cattle, it started at amino acid position 1781 and ended at 3868. From the analysis of alignment data for other sequences obtained from NCBI BLAST, the L segment for 201808HABDVS from sheep had a 99.90% identity with sequences of RVFV isolate 201,902,750 all from Uganda, while the lowest percentage identity was 98.43% with RVFV isolate VRL 1217/78 L segment complete sequences from South Africa. The 201810CML3DVS from cattle had a highest percentage identity of 99.97% with RVFV isolate 2,019,000,825 segment complete sequences from Uganda and a lowest percentage identity of 98.52% with RVFV isolate SPU142/08 L segment complete sequence from South Africa.

Phylogenetic tree for L Segment Fig. S5 constructed from a total of 37 sequences of different lineages from NCBI Genbank showed both isolates OP158214 and OP158211 both highlighted in yellow belong to lineage C and clustered with other isolates from previous outbreaks determined to be in lineage C.

### Identification of S, M, and L genome segment recombinants of RVFV strains isolated from cattle, sheep, and goat serum samples

After setting the parameters as required in RDP version 5, the results indicated no evidence of recombination by showing a total of zero recombination signals in the S, M, and L complete genome sequences.

### Detection of natural reassortment events in the isolated S, M, and L segments

Phylogenetic analysis of the S, M, and L genome segments of the RVFV isolates in this study highlighted in yellow in Figs. S3, S4 and S5 showed that they clustered differently from the same selection of other RVFV sequences selected from GenBank (Figs. S3, S4, and S5). The S, M and L-segments of the 201808HABDVS OP158209, OP158213 and OP158211respectivelyfrom sheep and the 201810CML3DVS OP158212, OP158210 and OP158214 respectively from cattle isolated in this study were of the same lineage C and clustered with other virus isolates in lineage C. Thus, the M-segment of 201808HABDVS OP158213 from sheep clustered alone after third speciation while that of OP158210 clustered alone after the fifth speciation within lineage C.

## Discussion

### Active Rift Valley fever virus infection in cattle, sheep, and goats during the interepidemic period

This study shows endemic infection of the RVFV in various regions of Kenya during interepidemic periods when there are no reported epidemics or outbreaks. The maintenance of RVFV between epidemics is still not completely understood; the general view has always been that the virus is maintained through vertical transmission within its arthropod vector, and wildlife are considered possible reservoirs [27, 28]. Above-normal and persistent rain leads to a rise in transmission pressure due to increased vector population densities. Travel, trade and tourism activities can facilitate long range transmission of the virus as the ease of air travel enable acutely infected individuals to rapidly reach new destinations [8]. In this study, 73% of the RVF cases were reported in the periods immediately following the conclusion of rainy seasons. Long rainy seasons are often observed between mid-March to May and a shorter period occurs in November and December.

During the study period, there was only one outbreak between June 2018 and September 2018, where the Government of Kenya, through the Directorate of Veterinary Services (DVS), declared a RVF outbreak in selected counties within Kenya. The two RVFV isolates fully sequenced in this study were both collected in early June 2018 at the onset of the outbreak from regions in northern Kenya. The isolates 201808HABDVS from sheep and 201810CML3DVS from cattle were isolated from Habaswen in Wajir County and Ittir village in Marsabit County (Fig. S1), respectively. Both were in the northern regions of Kenya, which were the epicentres of the 2018 RVF outbreak. But before the June 2018-September 2018 outbreak, the last major outbreak happened in 2006–2007. However, the isolates in this study did not show direct lineage from the Kenyan isolates of the same period as the Sudan outbreak. Species positivity rate was 29.2% for cattle, 30.8% for goats and 29.3% for sheep. The exposure levels were comparable and this could be due to similarities in exposure conditions as the sampled in animals were raised in the same ecological regions.

### Phylogenetic analysis of the Rift Valley fever virus genome

Phylogenetic methods were used to study the full genome sequences of the three segments of the two RVFV isolates to figure out their relationship with other RVFV strains that have been found in the past. The phylogeny of the S, L and M segments of the two virus isolates showed they were all of lineage C and in all the three phylogenetic trees (Figs. S3-S5), the segments clustered together with other virus isolates from previous outbreaks in lineage C. This strongly indicates that RVFV continues to spread to other regions across the globe from its epicenter the Rift Valley region of Kenya where it was first reported in 1931 [29, 30]. The sharing of ancestry with other viruses though of the same lineage but from various outbreaks and different countries suggests a slow evolution clock in the RVFV and suggests the two virus strains had different origins.

### Recombination of the S, M, and L genome segment recombinants of the Rift Vlley fever virus genome

Viral recombination occurs when viruses of two different parent strains co-infect the same host cell and undergo genetic crossing-over during replication to generate virus progeny that have some genes from both parents on the same genome strands. While it was expected that recombination events would be observed in the M segments that code for the surface glycoproteins compared with the S and L genome segments due to selective pressure from neutralizing antibodies, this was not the case. In this study, there was no evidence of recombination, possibly because of factors like genetic, biological, and epidemiological factors that can affect the probability of recombination between different RVFV strains, including genetic homology, population size in the host, co-circulation in the same geographical area, prevalence in the population, and low co-infection rate [31–33]. Also, RVFV is ( −) ssRNA virus. These viruses have a stable genome and show the lowest rates of recombination [34]. Lack of recombination could also be due to high conservation of the RVF genome sequence, which suggests a slow evolutionary clock, low tolerance to recombination, or that the isolates have a relatively recent common ancestor [35, 36]. Some previous studies on the characterization of the RVFV S, M and L genome segments have also found no natural reassortment in the RVFV S, M and L genome segments [37]. In general, the RVFV genome is characterized by low genetic diversity (~ 5%); consequently, it is difficult to statistically detect intragenic recombination events [38].

### Reassortment

Genomic surveillance has become an important technique for studying emerging and re-emerging infectious diseases so as timely interventions are instituted in its treatment, prevention and vaccine development if applicable. It was very important that reassortment events are checked so that the actual cause of the change in virulence can be ascertained. Phylogenetic trees were constructed using Maximum parsimony method at 1000 iterations, the phylogenetic relationship of the 2 RVFV isolates was assessed in relation to other RVFV isolates of different lineages whose genome sequences were already in GenBank. We checked for reassortment of the two RVFV isolates genome sequences for S, M and L segments. Using the two RVFV as the references one at a time in NCBI BLAST the search was set at maximum target of 5000 hits with an expect threshold value (E) at 15. Sequences were selected based on percentage identity and also ensuring all the lineages A-O were included. Lineages were determined using data from previous studies [39–41] and also by use of a computational method presented both as a command line tool and a web application hosted at https://www.genomedetective.com/app/typingtool/rvfv/ [42]. Phylogenetic trees for the three genome segments were prepared using Maximum Parsimony at 1000 iterations. All the three whole genome sequence segments (S, M and L) for the two RVF viruses 201808HABDVS and 201810CML3DVS clustered within the lineage C. There was no evidence of a possible reassortment event in all the three segments from the two virus isolates as none of the segments clustered with other segments outside the lineage C. This is very likely because sequencing and phylogenetic studies conducted over the last 20 years have shown the RVFV genome to be overall highly conserved, with most variances occurring as random single site mutations throughout the genome with no well-defined variable regions [43]. These findings also show that it is not only reassortment which causes change in the virus virulence but there could be other likely factors possibly (environmental, host, vector and virus) which could possibly lead to change in the pathogenicity and virulence of the RVFV.

The majority of reassortment events described in RVFV involve the exchange of segment M, resulting in an antigenic shift due to the two glycoproteins Gn and Gc encoded by this segment [44].

## Conclusion

This study identified active cases of RVF during an interepidemic period, which under normal circumstances would be given little or no attention. However, characterization of the isolated RVFV genomes showed that the virus remained genetically active during this period. There was no statistically significant evidence of recombination in any of the three genome segments of the two virus isolates and phylogenetic analysis showed no interlineage clustering hence no reassortment of the L, M and S segments findings that are in agreement with previous findings that the RVFV genome is stable and reassortments are rare. This strongly suggests that vector, host and environmental factors could be contributing to the emergence of virulent strains.

While this study was successful, we encountered some limitations;

1. Only IgM positive samples were archived hence we were not able to isolate the virus from the IgM negative samples by cell culture since they were not available
2. Serum samples were used to detect and isolate the virus and are less sensitive compared to using whole blood in virus isolation and detection.

### Supplementary Information

Supplementary Material 1.

## Data Availability

Accession numbers for the isolated genome sequences data are OP158209 - OP158214 and have been deposited in the NCBI Genbank.
