# Supplementary material for: Genome characterization of Rift Valley fever virus isolated from cattle, goats and sheep during interepidemic periods in Kenya
Source: BMC Vet Res. 2024 Aug 23;20:376. doi: 10.1186/s12917-024-04161-1 (PMC11342565; doi:10.1186/s12917-024-04161-1)
Supplement: Supplementary file 1 — Supplementary Material 1. [file 12917_2024_4161_MOESM1_ESM.docx]

**APPENDICES**

**Table S1.** Primers and probe used to detect the RVFV L-segment by RT-qPCR (21).

| **Type/Sub type** | **Gene** | **Primer** | **Sequence 5' - 3'** |
| --- | --- | --- | --- |
| Rift Valley fever virus | L segment | RVFL-2912fwdGG | TGAAAATTCCTGAGACACATGG |
| Rift Valley fever virus | L segment | RVFL-2981revAC | ACTTCCTTGCATCATCTGATG |
| Probe | L segment | RVFL-probe2950 | CAATGTAAGGGGCCTGTGTGGACTTGTG |

**Table S2.** The GenBank accession numbers of the complete genome sequences of two RVFV strains.

| **Sample Identification** | **Genome Segment Identification** | **GenBank Accession Number** |
| --- | --- | --- |
| 201808HABDVS | 2609218 08HAB_S | OP15820 |
|  | 2609218 08HAB_M | OP158210 |
|  | 2609218 08HAB_L | OP158211 |
| 201810CML3DVS | 2609218 CML3_S | OP158212 |
|  | 2609218 CML3_M | OP158213 |
|  | 2609218 CML3_L | OP158214 |

**
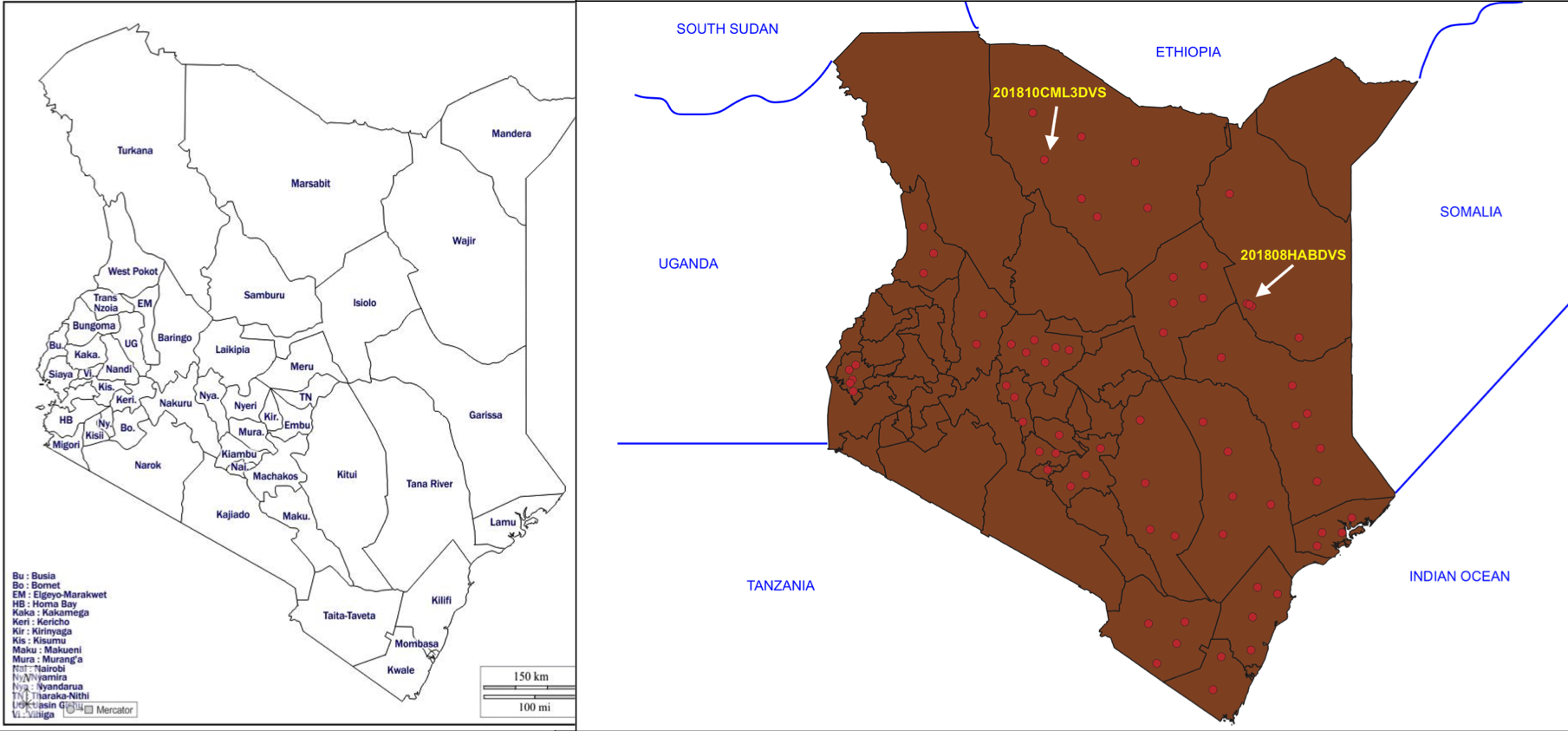
**

**Figure S3.** An administrative map of Kenya showing the county locations (left panel) and the sampling points (right panel). The two RVFV isolates that were sequenced are highlighted on the right panel, and the locations from where they were obtained are indicated with the arrows.

**
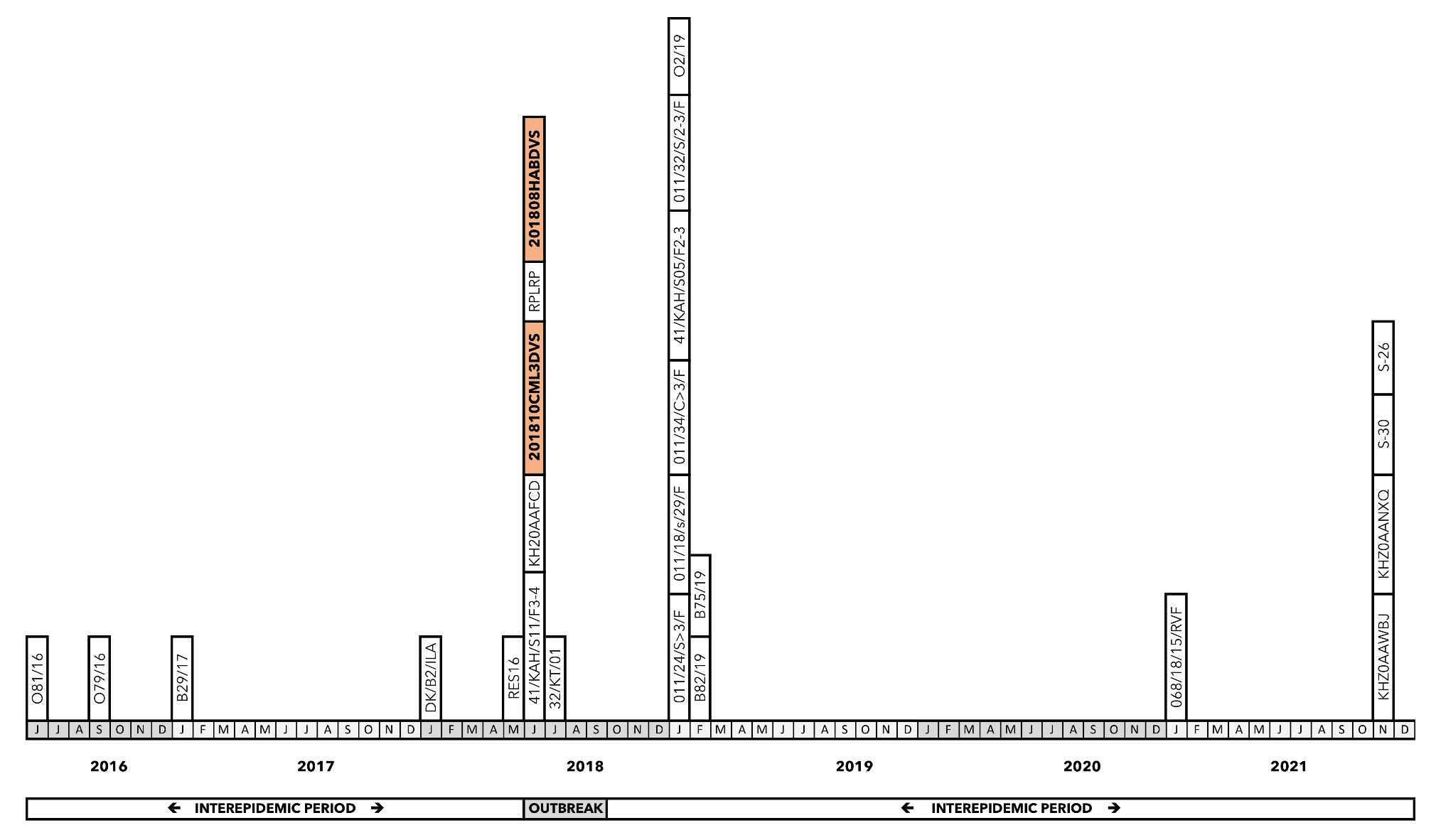
**

**Figure S4.** The 24 RT-qPCR positive samples mapped to the study window that started in June 2016 and ended in November 2021. There were two interepidemic periods, immediately before and immediately after a short 4-month outbreak. In this study, 73% of the RVF cases were reported in the periods immediately following the conclusion of rainy seasons, the heaviest falling from mid-March to May and a shorter period of rain occurs in November and December.


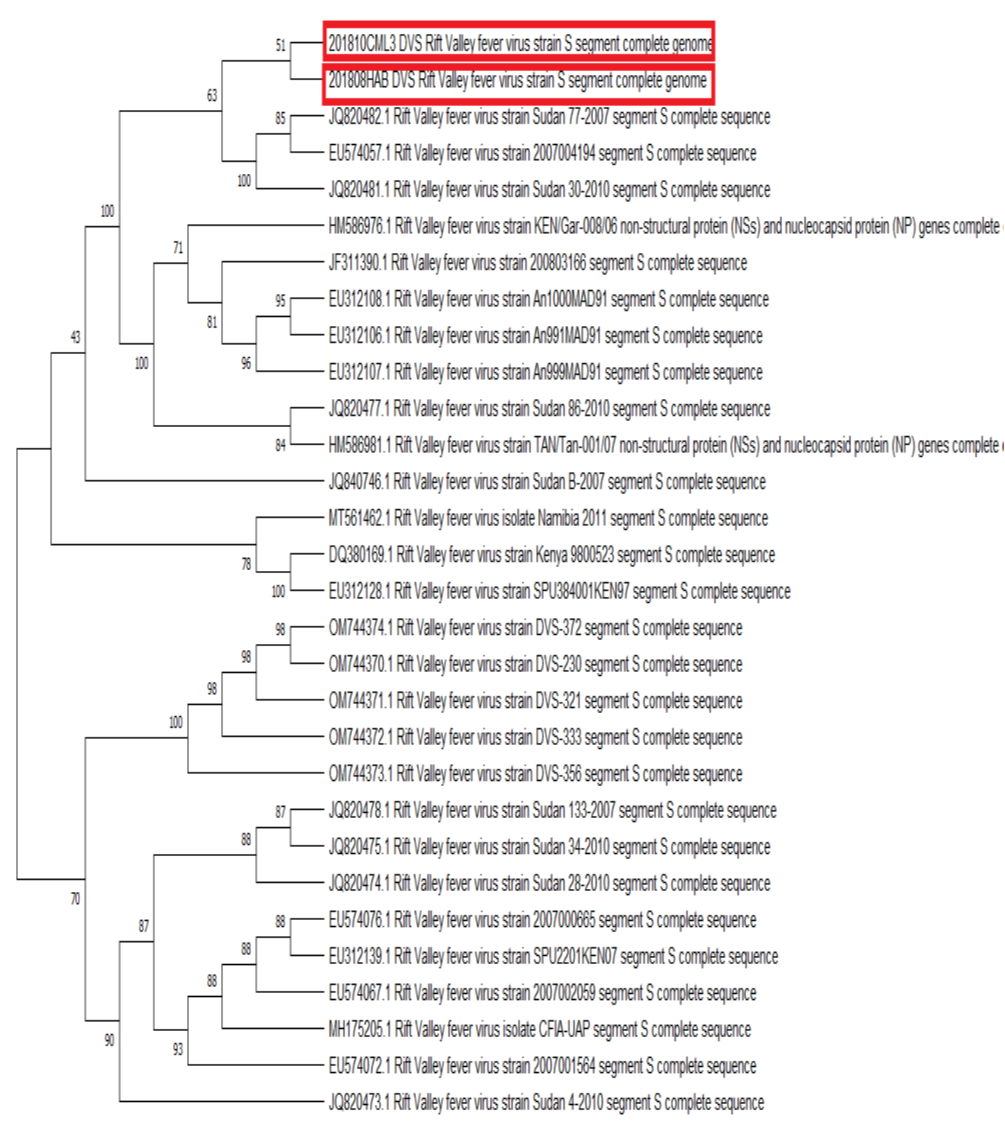


**Figure S5.** Phylogenetic tree for the S genome segment. Maximum parsimony method at 1000 iterations using MEGA version 11. With a bootstrap value of 51.0%, the S genome segments 201810CML3DVS from cattle and 201808HABDVS from sheep were found to be in the same clade. They shared the same ancestry with the clade of RVFV strain 2007004194 (GenBank accession number EU574057) and RVFV strain Sudan 77-2007 (GenBank accession number JQ820482).


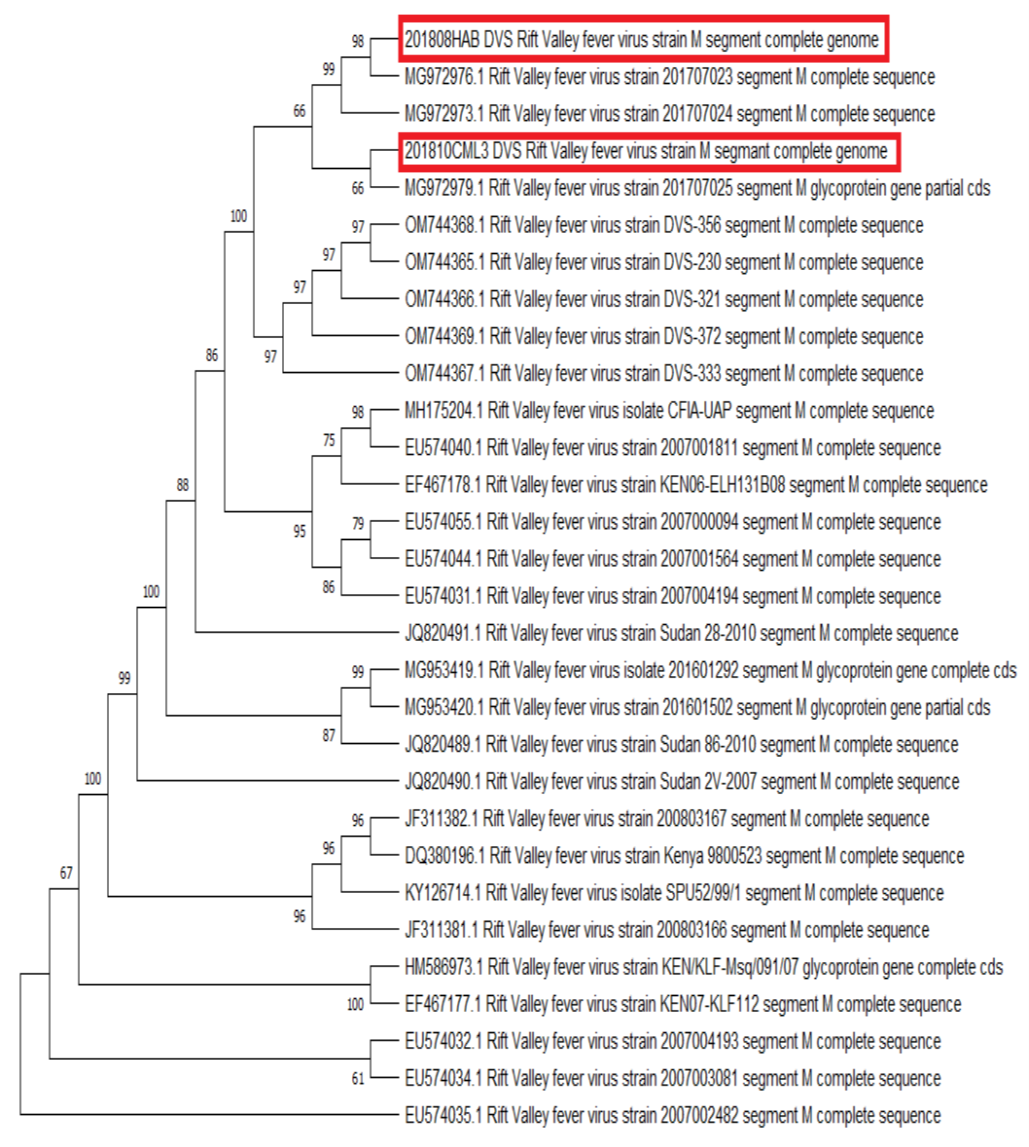


**Figure S6.** Phylogenetic tree of the M segment by the maximum parsimony method at 1000 iterations using MEGA version 11. The M segment of the 201810CML3DVS strain from cattle clustered in the same clade with the RVFV strain 201707023 M segment (GenBank accession number MG972976) at a bootstrap value of 98.0%. This clade also shared the same ancestry with the RVFV strain 201707024 M segment (GenBank accession number MG972973) at a bootstrap value of 99.0%.


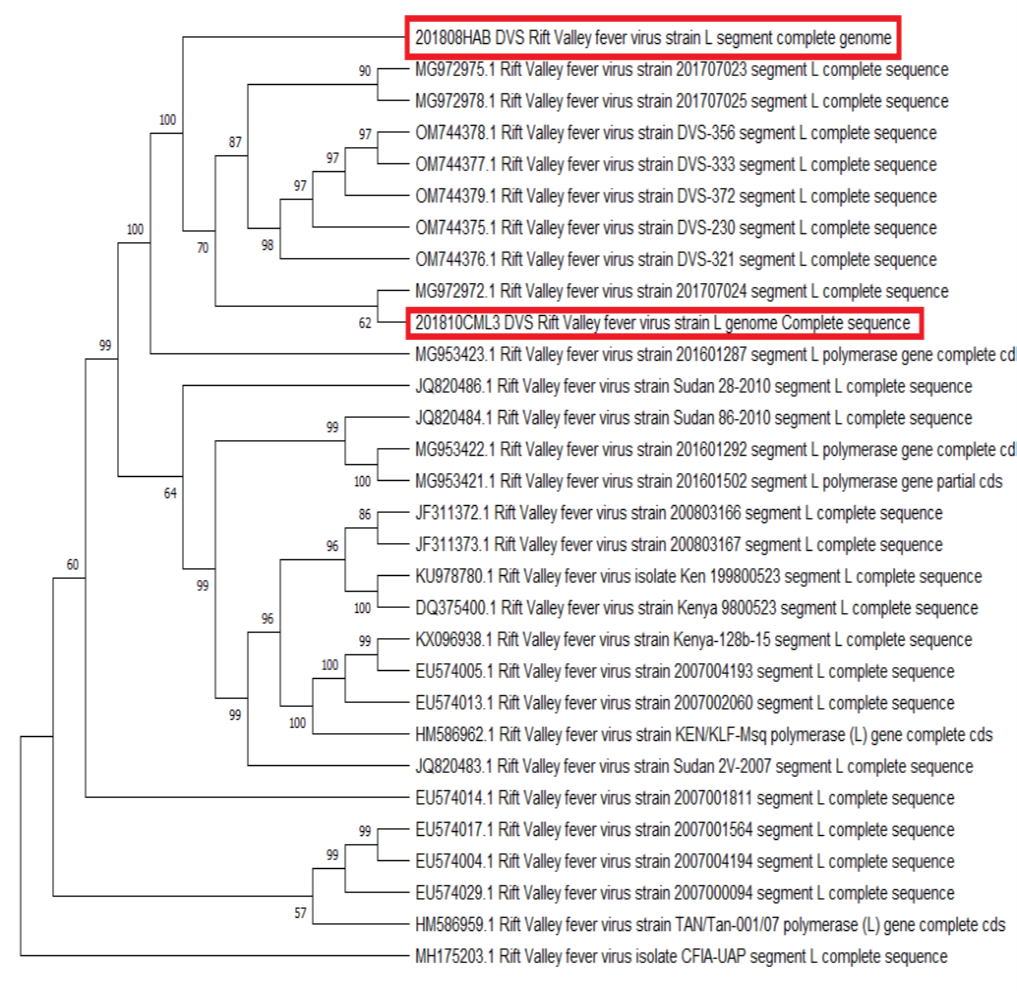


**Figure S7**. Phylogenetic tree for the L segment by maximum parsimony at 1000 iterations using MEGA version 11. The RVFV L segment of the 201810CML3DVS isolate from cattle clustered in the same clade as the Rift Valley fever virus strain MG972972 version MG972972.1, and the 201810CML3DVS L segment is distantly related to that of the 201808HABDVS isolate from sheep.
